# Efficient Regeneration of Degraded LiNi_0.9_Mn_0.1_O_2_ by Acid Etching–Hydrothermal Relithiation Coupled with Li_4_Ti_5_O_12_ Coating

**DOI:** 10.3390/nano16100585

**Published:** 2026-05-11

**Authors:** Jiwei Hao, Longwei Liang, Jiawei Mu, Zhenyuan Xie, Hongqiang Xi, Linrui Hou, Changzhou Yuan

**Affiliations:** School of Materials Science & Engineering, University of Jinan, Jinan 250022, China

**Keywords:** spent lithium-ion batteries, high-nickel cathode materials, direct regeneration, single-crystallization, LTO coating modification

## Abstract

With the growing global demand for sustainable resources, recycling spent lithium-ion batteries has become a strategic priority. Conventional pyrometallurgical and hydrometallurgical methods suffer from high energy consumption, severe pollution, and structural destruction, making them unsuitable for regenerating high-nickel cathodes. In this work, spent polycrystalline high-nickel LiNi_0.9_Mn_0.1_O_2_ cathodes were selected, and an upcycling strategy integrating acid etching, hydrothermal relithiation, short-time annealing, and simultaneous Li_4_Ti_5_O_12_ (LTO) coating was developed. This process directly transformed degraded polycrystalline cathodes into single-crystal cathode materials with excellent structural stability and electrochemical performance. During regeneration, lithium compensation and lattice recrystallization effectively repaired lithium loss, reduced Li/Ni cation mixing, reactivated the degraded structure, and reconstructed a highly ordered layered single-crystal framework. The LTO coating further stabilized the cathode/electrolyte interface, suppressed side reactions, alleviated volume strain, and promoted Li^+^ transport kinetics. Electrochemical measurements showed that the regenerated single-crystal cathode exhibited superior structural integrity, strong resistance to crack propagation, low polarization, excellent rate capability, and long-term cycling stability. A capacity retention of 84.3% was achieved after 300 cycles at 1C, outperforming commercial polycrystalline cathodes. This strategy provides an efficient and promising route for the direct regeneration of spent high-nickel ternary cathodes.

## 1. Introduction

Over the past decade, layered ternary oxide cathode materials have become the mainstream solution for energy storage in portable electronics and electric vehicles because of their high energy density and long cycle life [1,2,3]. However, the limited service life of batteries, typically 5–8 years [4], has led to an increasingly severe challenge in the disposal of spent lithium-ion batteries. Critical elements in lithium-ion batteries, such as Li, Co, and Ni, are extremely limited and unevenly distributed worldwide [5], with lithium and cobalt resources in particular being concentrated in only a few countries and regions. Therefore, end-of-life batteries present a dual challenge: they pose environmental risks while also representing valuable secondary resources. On the one hand, these batteries contain toxic and flammable components as well as heavy metals such as Ni, Co, Mn, Li, Fe, Cu, and Al, and thus must be handled with great caution during disposal [6]. Improper treatment, such as landfilling or incineration, may result in serious environmental contamination by allowing pollutants to leach into soil and water systems [7]. On the other hand, the rational recycling and reutilization of such batteries can generate substantial resource, economic, and environmental benefits. At present, pyrometallurgical and hydrometallurgical processes remain the two most important approaches for lithium battery recycling and have both reached a mature stage of industrial development [8]. However, pyrometallurgy is highly energy-intensive, whereas hydrometallurgy requires large amounts of chemical reagents. Moreover, both routes involve significant environmental risks and can no longer fully satisfy the demands of modern sustainable development [9,10].

Direct recycling technology has attracted increasing attention as a potential alternative to these conventional methods, because it can repair structural defects and convert end-of-life cathode materials back into battery-grade cathodes without destroying their original framework [11,12]. Various direct regeneration methods, including solid-state sintering [13], pressurized hydrothermal treatment [14] and eutectic salt processing [15], have been developed. These methods generally rely on chemical relithiation combined with annealing to restore the composition and structure of degraded materials [16]. Nevertheless, when applied to high-nickel ternary cathodes with Ni content ≥ 0.9, substantial challenges remain. High-nickel ternary cathodes are widely recognized as key cathode materials for next-generation high-energy-density lithium-ion batteries because of their high specific capacity [17]. However, with increasing Ni content, these materials suffer from more pronounced capacity fading and poorer thermal stability [18]. These problems originate from active lithium loss and multiple phase transitions and are also closely associated with coupled degradation mechanisms such as lattice oxygen release, Li/Ni cation mixing, particle microcrack propagation, and transition-metal dissolution [19]. Hydrothermal relithiation has recently attracted attention for the direct regeneration of high-nickel cathodes because it can precisely and uniformly replenish the active lithium lost from spent cathodes while avoiding prolonged solid-state sintering [20]. Several research groups have developed direct regeneration strategies combining hydrothermal relithiation with short-time annealing, successfully regenerating spent LiCoO_2_ [21], LiNi_x_Co_y_Mn_1−x−y_O_2_ [22], and LiFePO_4_ cathodes [23], with the regenerated materials exhibiting electrochemical performance comparable to that of pristine cathodes. Hydrothermal relithiation can accurately repair lithium deficiency in cathodes regardless of differences in lithium loss among spent batteries, and when combined with short-time annealing, it restores the material to the desired stoichiometry and crystallinity. Notably, the subsequent annealing step requires only 4 h, far shorter than the >10 h high-temperature sintering typically required for conventional solid-state synthesis of cathodes, thus offering a simpler process and lower energy consumption [24].

Furthermore, all degradation behaviors of NCM cathodes can be directly attributed to an unstable cathode/electrolyte interface [25]. Therefore, recent studies on high-nickel NCM materials have shown that surface coating is a simple yet effective strategy for improving electrochemical performance [26]. Coatings can play multiple roles in stabilizing cathode surfaces and prolonging cycle life. For example, the typical fast lithium-ion conductor Li_4_Ti_5_O_12_ (LTO) has been widely reported as an effective functional coating [27]. Its spinel structure provides lithium-ion migration pathways, while the octahedral and tetrahedral interstitial sites in the lattice enable rapid Li^+^ transport [28]. Accordingly, an LTO coating can not only improve Li^+^ diffusion kinetics but also buffer the direct contact between layered oxide materials and the electrolyte. In addition, as a zero-strain material, LTO can significantly improve electrochemical performance by alleviating the volume change of NCM materials during Li^+^ intercalation/deintercalation. It is also worth noting that the introduction of coating materials, including LTO, during regeneration should help repair microcracks in degraded NCM materials, thereby enhancing the integrity of the regenerated particles [29].

Accordingly, in this work, we developed a strategy that integrates acid etching, hydrothermal relithiation, and short-time annealing to directly convert spent polycrystalline Ni-rich cathodes into single-crystal cathode materials with uniform particle morphology. The acid-etching process dissociates degraded polycrystalline particles, followed by hydrothermal treatment for elemental compensation and surface coating, and then a mild annealing step to restore the LiNi_0.9_Mn_0.1_O_2_ (NM91) phase structure and crystallinity. The results show that a uniform LTO coating layer is formed on the surface of the upcycled particles, endowing the regenerated material with outstanding electrochemical performance that even surpasses that of commercial polycrystalline cathodes. This upcycling strategy should be broadly applicable to other types of degraded NCM materials and may provide important insights for a technological revolution in spent lithium-ion battery recycling, thereby contributing to a greener future.

## 2. Experimental Section

### 2.1. Pretreatment of Spent LIBs

Commercial polycrystalline LiNi_0.9_Mn_0.1_O_2_ (C-NM91) was purchased from Canrd Technology Co., Ltd. (Dongguan, Guangdong, China). The spent polycrystalline LiNi_0.9_Mn_0.1_O_2_ (S-NM91) was obtained by disassembling pouch cells assembled with C-NM91 after long-term cycling. Before disassembly, the spent batteries were fully discharged in an aqueous NaCl solution. After discharge, the batteries were manually dismantled in a fume hood to recover the cathode foils. The foils were immersed in dimethyl carbonate (DMC) for 5 h to remove residual electrolyte, and then ultrasonic treatment was performed for 5 to 10 min to effectively separate the spent polycrystalline LiNi_0.9_Mn_0.1_O_2_ (S-NM91) active material from the aluminum foil. The recovered S-NM91 powder was calcined at 500 °C for 4 h in a tube furnace under air atmosphere to remove the PVDF binder and acetylene black impurities. Under this thermal condition, PVDF decomposes into HF, fluorocarbons, and carbon oxides, while acetylene black is fully oxidized to CO_2_ [30,31,32]. Considering the corrosiveness of HF generated during PVDF decomposition, an alumina (Al_2_O_3_) tube was adopted for the calcination process to ensure safety and stability.

### 2.2. Regeneration of R-NM91@LTO

The spent S-NM91 cathode material was treated with 0.5 mol L^−1^ dilute sulfuric acid at a solid-to-liquid ratio of 0.05 g mL^−1^. The mixture was continuously stirred on a magnetic stirrer for 3 h and then separated by centrifugation. The collected solid was repeatedly washed with deionized water to remove residual acid and then dried at 60 °C. A 5% excess amount of LiOH, calculated based on the mass of acid-treated E-NM91 powder, was mixed with the acid-treated cathode material and homogenized by ball milling. The resulting mixture was dispersed in an aqueous solution at a mass ratio of 1:10, followed by ultrasonication for 30 min to ensure uniform dispersion. Subsequently, 1% tetrabutyl titanate was added, and NH_3_·H_2_O was introduced dropwise to adjust the pH to approximately 10–11. The mixed solution was then transferred into a Teflon-lined stainless-steel autoclave and heated at 220 °C for 12 h, followed by solid–liquid separation by centrifugation. The collected solid was repeatedly washed with deionized water to remove residual LiOH and then dried in an oven at 60 °C. The dried mixture was finally annealed in a tube furnace under an oxygen atmosphere by heating to 750 °C at 5 °C min^−1^ and holding for 4 h, this annealing condition was optimized to fully restore the layered crystal structure, promote sufficient crystallization, and ensure the complete formation of the LTO coating without causing obvious particle sintering or agglomeration, yielding the regenerated material R-NM91@LTO (regenerated LiNi_0.9_Mn_0.1_O_2_@Li_4_Ti_5_O_12_).

### 2.3. Materials Characterization

Crystal structures of the samples were characterized by X-ray diffraction (XRD, Rigaku Ultima IV, Hokuto, Yamanashi, Japan) using Cu Kα radiation (λ = 1.5046 Å) at 40 kV and 120 mA. Data were collected in the 2θ range of 10–80° at a scanning rate of 10° min^−1^. Rietveld refinement of the lattice parameters was performed using the GSAS/EXPGUI software package (Version 3.0). Chemical states and elemental composition were analyzed by X-ray photoelectron spectroscopy (XPS, Thermo ESCALAB 250Xi, East Grinstead, West Sussex, UK) with a monochromatic Al Kα source. All spectra were calibrated using the C 1s peak at 284.8 eV. Morphology and elemental distribution were examined using field-emission scanning electron microscopy (FESEM, Zeiss Gemini 300, Oberkochen, Germany) operated at 15 kV. Microstructural analysis was conducted by transmission electron microscopy (TEM) and high-resolution TEM (HRTEM) on a JEOL JEM-2100 system operating (Akishima, Tokyo, Japan) at 200 kV. For post-cycling characterization, electrodes retrieved from disassembled cells were gently ground and thoroughly rinsed with DMC in an argon-filled glove box to remove residual electrolyte, followed by drying under an inert atmosphere.

### 2.4. Electrochemical Measurements

As for half-cell assembly, cathode electrodes were fabricated by coating a slurry of active material, polyvinylidene fluoride (PVDF) binder, and carbon black (8:1:1 by weight) in N-methyl-2-pyrrolidone (NMP) onto aluminum foil. The areal mass loading of active material for all fabricated electrodes was controlled at approximately 3.0 ± 0.1 mg cm^−2^. The electrodes were then dried under vacuum at 110 °C for 12 h. Coin-type half-cells (2032) were assembled in an argon-filled glove box (O_2_ and H_2_O < 0.1 ppm) using lithium foil as the counter/reference electrode, a Celgard 2400 separator, and 1 M LiPF_6_ in ethylene carbonate/dimethyl carbonate/ethyl methyl carbonate (EC/DMC/EMC, 1:1:1 by volume) as the electrolyte. Cells were cycled between 2.8–4.3 V (vs. Li/Li^+^). The initial three formation cycles were performed at 0.1C (1C = 200 mAh g^−1^), followed by long-term cycling at 1C. Rate capability was evaluated at current rates ranging from 0.1 to 5C. All galvanostatic charge–discharge tests were conducted using a Land test system (CT2001A, Wuhan, China). Galvanostatic intermittent titration technique (GITT) measurements were performed on the Land system to assess electrode kinetics. Each GITT cycle consisted of a 10 min charging pulse at 0.1C followed by a 20 min relaxation period. Electrochemical impedance spectroscopy (EIS) was measured at 4.6 V (vs. Li/Li^+^) over a frequency range of 100 kHz to 5 mHz with an AC amplitude of 1 mV. Cyclic voltammetry (CV) and EIS tests were recorded with an IVIUM electrochemical workstation (Ivium Technologies BV, Eindhoven, The Netherlands).

## 3. Result and Discussion

**Figure 1:**
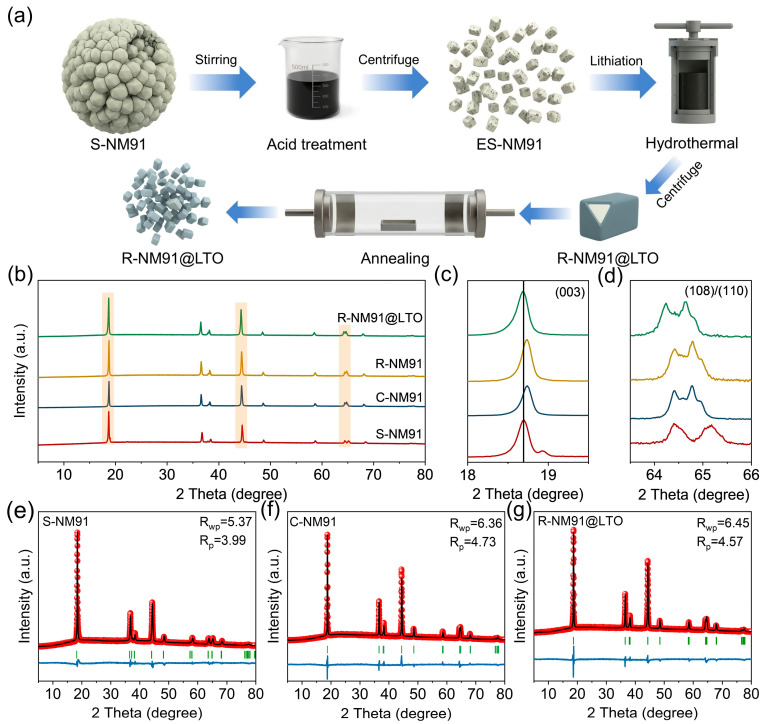
(**a**) Schematic illustration for the proposed regeneration route. (**b**) X-ray diffraction (XRD) patterns of S-NM91, C-NM91, R-NM91 and R-NM91@LTO. Enlarged XRD patterns of (**c**) (003) and (**d**) (108)/(110) peaks of S-NM91, C-NM91, R-NM91 and R-NM91@LTO. XRD patterns and Rietveld refinement files of (**e**) S-NM91, (**f**) C-NM91 and (**g**) C-NM91.

In this work, we propose a regeneration strategy that combines acid etching with hydrothermal treatment and short-time annealing (Figure 1a)**,** enabling the direct restoration of severely degraded polycrystalline high-nickel S-NM91 into structurally stable single-crystal R-NM91@LTO with enhanced performance. Specifically, when the degraded polycrystalline Ni-rich cathode is dispersed in dilute sulfuric acid, the combined effects of acid corrosion and external mechanical agitation gradually break the intergranular bonding within the secondary particles, causing them to dissociate into individual single-crystal primary particles [33]. During the acid-etching process, all surface-bound impurities are effectively removed while the active layered phase is retained. This phase possesses intrinsic lithium-ion conduction channels that facilitate Li^+^ transport and permit lithium replenishment during the subsequent solid-state sintering step. The acid-etched but unregenerated intermediate product obtained in single-crystal form is denoted E-NM91 (Figure S1). If conventional solid-state relithiation is applied to E-NM91, its excessively small particle size tends to lead to obvious aggregation in the final product. To overcome this limitation, we proposed a mild hydrothermal relithiation process, during which a lithium titanate precursor was simultaneously introduced, followed by a short-time solid-state annealing step to generate a uniform, continuous, and tightly bonded LTO coating layer, ultimately producing R-NM91@LTO with a uniform particle size distribution.

First, the crystal structure evolution induced during regeneration was investigated by X-ray diffraction (XRD). Figure 1b presents the XRD patterns of S-NM91, C-NM91, the regenerated sample R-NM91 obtained by hydrothermal treatment followed by annealing, and the LTO-coated regenerated sample R-NM91@LTO. All samples exhibit the typical α-NaFeO_2_ layered structure belonging to the *R-3m* space group. Notably, the (003) peak of S-NM91 shifts toward a lower diffraction angle (Figure 1c), which can be attributed to enhanced electrostatic repulsion between adjacent oxygen layers driven by lithium loss at the 3a site [34,35]. In addition, the broadening of the (108)/(110) doublet in S-NM91 indicates the transformation of the original layered structure into a detrimental rock salt phase, leading to phase distortion (Figure 1d) [36,37]. In sharp contrast, the (003) peak of R-NM91 shifts back toward a higher angle and approaches the position of the (003) peak of C-NM91, indicating that the layered structure has been effectively restored [38,39]. However, after LTO coating, the (003) peak shifts again slightly toward the lower-angle region, which may be explained by partial Ti^4+^ doping into the surface of the NCM particles, resulting in an enlarged lithium-layer spacing [34]. Moreover, compared with S-NM91, the splitting of the (108) and (110) peaks in R-NM91@LTO becomes slightly less pronounced, while the peak intensity increases significantly, indicating successful recovery of the layered crystal structure.

To obtain more precise crystallographic information, Rietveld refinement was performed on the XRD patterns of all samples (Figure 1e–g). As shown in Table S1, S-NM91 exhibits significant Li/Ni cation mixing (9.97%) and an apparently enlarged c lattice parameter (14.4124 Å), both of which are associated with lithium loss. In contrast, the cation disorder and lattice distortion in regenerated R-NM91@LTO are markedly alleviated, decreasing to 4.11% and 14.2163 Å, respectively, further confirming the successful restoration of a highly ordered layered structure.

The ICP-OES results (Table S3) provide quantitative evidence for the elemental composition changes during the regeneration process. For the spent cathode S-NM91, the Li molar ratio is only 0.881, indicating severe Li loss during cycling, while the Ni/Mn ratio remains close to the nominal 0.9/0.1 stoichiometry. In contrast, the commercial sample C-NM91 shows a Li molar ratio of 1.011, consistent with the designed LiNi_0.9_Mn_0.1_O_2_ composition. Notably, the regenerated sample R-NM91@LTO exhibits a Li molar ratio of 1.007, which is nearly identical to that of C-NM91. Meanwhile, the Ni/Mn ratio of R-NM91@LTO (0.891/0.109) is also consistent with both C-NM91 and the theoretical stoichiometry. These results confirm that our regeneration strategy successfully restores the Li stoichiometry of the spent cathode without altering the bulk Ni/Mn composition, yielding a regenerated material with elemental composition comparable to the commercial counterpart.

**Figure 2:**
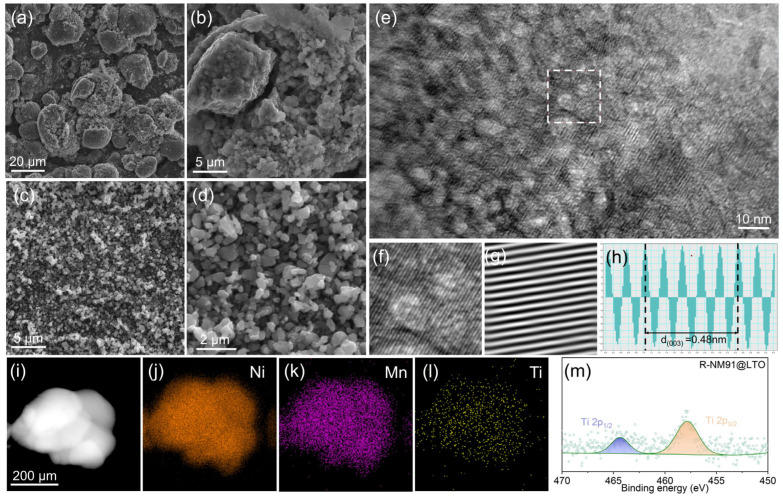
Field-emission scanning electron microscopy (FESEM) images of (**a**,**b**) S-NM91 and (**c**,**d**) R-NM91@LTO; (**e**–**h**) HRTEM images, magnified views, corresponding FFT spectra and lattice spacing diagrams of R-NM91; (**i**–**l**) EDS images of R-NM91@LTO; (**m**) Ti 2p spectrum of R-NM91@LTO.

The entire regeneration process was further elucidated through detailed analysis of particle morphology and microstructural evolution. Field-emission scanning electron microscopy (FESEM) images of S-NM91 and R-NM91@LTO are presented in Figure 2. Figure 2a,b shows the morphology of the spent polycrystalline cathode S-NM91, which displays obvious aggregation, cracking, and structural damage. The particles are rough and irregular, and residual binder and electrolyte species remain on the surface. These features are typical manifestations of lattice distortion, interfacial separation, and structural collapse after prolonged cycling, directly leading to electrochemical deterioration [40]. In contrast, the regenerated R-NM91@LTO sample in Figure 2c,d exhibits a uniformly dispersed single-crystal morphology with homogeneous particle size, a dense and smooth surface, and no obvious agglomerates or microcracks. This is in sharp contrast to C-NM91 (Figure S2). To further probe the microcrystalline structure of R-NM91@LTO, the TEM image shown in Figure 2e reveals continuous and well-ordered lattice fringes, with no obvious lattice distortion or amorphous impurity regions, indicating excellent single-crystal orientation and crystallinity. This is further confirmed by the enlarged image (Figure 2f) and the corresponding FFT pattern (Figure 2g). In addition, the measured lattice spacing of d_(003)_ = 0.48 nm is in good agreement with the standard spacing of the (003) plane in the *α*-NaFeO_2_-type layered NCM structure, demonstrating that the recycling process restores not only the macroscopic morphology but also the layered crystal structure. The EDS elemental mapping images of R-NM91@LTO (Figure 2i–l) show that the transition metals Ni and Mn, as well as Ti, are uniformly distributed throughout the particles without obvious elemental segregation, indicating that the recycling process does not disrupt the chemical stoichiometry and that a stable and uniform LTO coating layer is formed on the particle surface. Furthermore, the Ti 2p XPS spectrum in Figure 2m clearly shows characteristic peaks at 458.5 and 464.2 eV, corresponding to Ti 2p_3_/_2_ and Ti 2p_1_/_2_ in LTO, directly confirming the successful formation of the surface LTO coating uniformly covering the single-crystal particles. Taken together, the SEM, TEM, and XPS results demonstrate that the recycling process successfully converts spent polycrystalline cathodes into single-crystal cathodes with intact structure and high crystallinity, while the uniformly distributed surface LTO coating further optimizes the interfacial state. This synergistic structural repair and interfacial regulation from the interior to the surface provides a robust microstructural and interfacial basis for the high specific capacity and long-term cycling stability of R-NM91@LTO.

**Figure 3:**
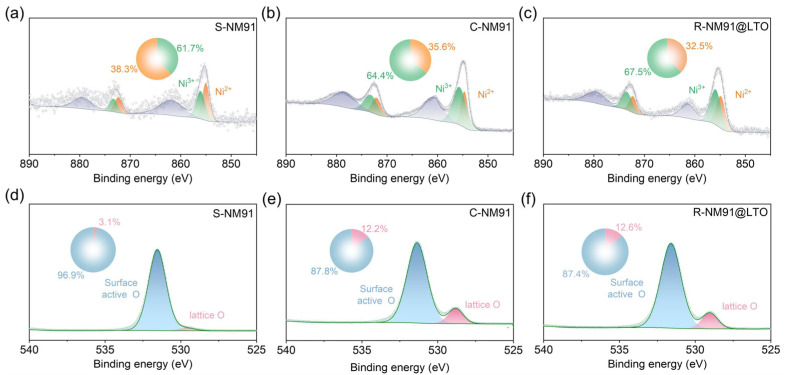
(**a**) Ni 2p spectra of S-NM91, (**b**) C-NM91 and (**c**) R-NM91@LTO; O 1s spectra of (**d**) S-NM91, (**e**) C-NM91 and (**f**) R-NM91@LTO.

X-ray photoelectron spectroscopy (XPS) was then employed to investigate the evolution of the Ni valence state and oxygen species during regeneration. Figure 3a–d presents the Ni 2p XPS spectra of the four samples. The proportion of Ni^3+^ in S-NM91 is only 38.3% (Figure 3a), much lower than that in C-NM91 (64.4%, Figure 3b), reflecting Ni reduction, valence-state disorder, and lattice degradation at the material surface after prolonged cycling [31,41]. After regeneration, the proportion of Ni^3+^ in R-NM91 increases to 57.1% (Figure S3), demonstrating that the recycling process effectively repairs the oxidation-state environment of Ni and suppresses surface reduction and dissolution [42,43]. After LTO coating, the proportion of high-valence Ni in R-NM91@LTO further increases to 67.5% (Figure 3d), and the peak shape becomes more regular, indicating that the LTO coating effectively stabilizes the surface chemical state of Ni and suppresses valence fluctuations caused by interfacial side reactions. The O 1s spectra further support these findings. As shown in Figure 3d–f, the O 1s spectra of all samples can be fitted with two components corresponding to lattice oxygen and surface-active oxygen. The proportion of lattice oxygen in S-NM91 is only 3.1% (Figure 3d), reflecting severe structural degradation characterized by oxygen vacancy enrichment and lattice oxygen loss after cycling [44], and is far lower than that in C-NM91 (64.4%, Figure 3e). After recycling, the lattice oxygen content increases to 11.7% and 12.6% in R-NM91 (Figure S4) and R-NM91@LTO (Figure 3f), respectively. The sharper and more symmetric lattice oxygen peaks indicate that the recycling process successfully restores the oxygen coordination environment and the integrity of lattice oxygen, while the LTO coating further optimizes the surface oxygen structure and reduces oxygen-vacancy-induced interfacial side reactions. Overall, the XPS analyses of Ni 2p and O 1s confirm that the recycling process effectively repairs the surface chemical state of spent S-NM91 by restoring the transition-metal valence states and lattice oxygen structure [45], and that the LTO coating further stabilizes the surface elemental states through interfacial regulation, suppresses transition-metal dissolution and electrolyte decomposition, and thereby provides a solid surface chemistry basis for the excellent electrochemical performance of R-NM91@LTO [46].

**Figure 4:**
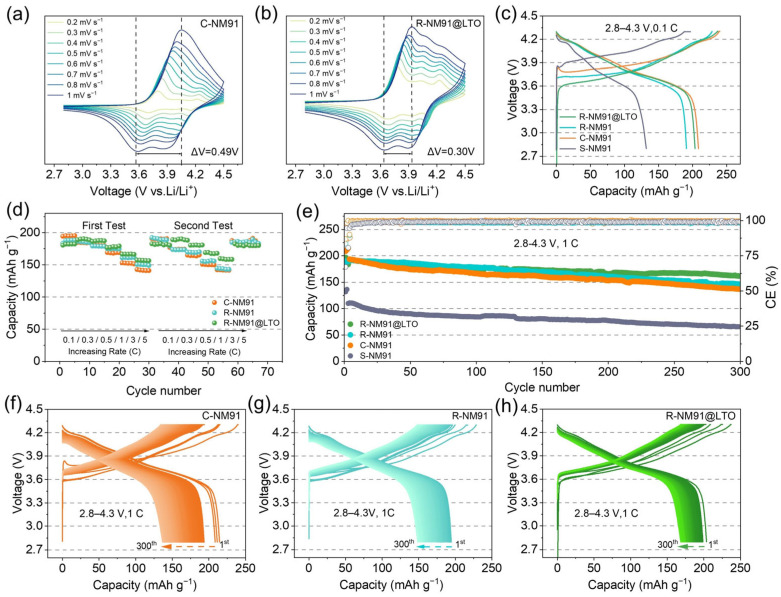
(**a**) Electrochemical evaluation in half cells of C-NM91, S-NM91, R-NM91 and C-NM91. Different CV scan rates of (**a**) C-NM91 and (**b**) R-NM91@LTO within 2.8–4.5 V, (**c**) Initial charge/discharge curves (0.1C), (**d**) Rate behaviors, (**e**) cycling stability within 2.8–4.3 V at 1C, and corresponding voltage profiles of (**f**) C-NM91, (**g**) R-NM91 and (**h**) R-NM91@LTO.

To evaluate the electrochemical performance of R-NM91@LTO, half-cells were assembled for testing. The cyclic voltammetry (CV) curves of the four samples were measured within 2.8–4.5 V at scan rates of 0.2–1 mV s^−1^. All samples display a pair of typical redox peaks corresponding to the reversible Ni^2+^/Ni^3+^/Ni^4+^ redox reactions in NCM cathodes. Among them, the spent polycrystalline S-NM91 (Figure S5) exhibits a high redox peak potential difference (ΔV) of 1.13 V, with broadened peaks and poor symmetry, indicative of severe electrochemical polarization and sluggish kinetics. This directly reflects the structural degradation and increased interfacial resistance after long-term cycling [47]. C-NM91 (Figure 4a) shows a ΔV of 0.49 V, significantly lower than that of the spent sample, although obvious peak shifts and peak broadening are still present. The regenerated single-crystal R-NM91 (Figure S6) exhibits a reduced ΔV of 0.42 V, with sharper and more symmetric redox peaks, demonstrating that the recycling process effectively repairs the crystal structure, lowers electrochemical polarization, and improves Li^+^ diffusion kinetics [48]. More importantly, the LTO-coated R-NM91@LTO shows the lowest ΔV of only 0.30 V among the four samples (Figure 4b), and its peak shape remains highly symmetric and stable at different scan rates. This directly confirms that the LTO coating further reduces the charge transfer resistance and significantly improves the reversibility and kinetics of Li^+^ insertion/extraction by optimizing the electrode/electrolyte interface. These CV results demonstrate that the synergistic modification induced by recycling reconstruction and LTO coating effectively suppresses electrochemical polarization and endows R-NM91@LTO with excellent reaction kinetics, thereby providing the basis for its superior rate capability.

Figure 4c shows the initial charge–discharge curves of the four samples at 0.1C within 2.8–4.3 V. The spent polycrystalline S-NM91 delivers an initial discharge specific capacity of only 132 mAh g^−1^, accompanied by rapid voltage platform decay and severe polarization. By contrast, the commercial polycrystalline C-NM91, regenerated single-crystal R-NM91, and LTO-coated R-NM91@LTO all exhibit initial discharge specific capacities close to 200 mAh g^−1^, together with more stable voltage plateaus and lower polarization, fully demonstrating the significant improvement in initial electrochemical performance achieved through the single-crystal structure and recycling process.

The excellent electrochemical performance of R-NM91@LTO is also reflected in its rate capability (Figure 4d). At low current rates (0.1–0.3C), the capacity of R-NM91@LTO is slightly lower than that of C-NM91. However, at 0.5C, the capacity of R-NM91@LTO exceeds that of C-NM91, and the capacity difference further widens as the rate increases. In particular, at 5C, R-NM91@LTO delivers a discharge capacity of 157.4 mAh g^−1^, which is significantly higher than that of C-NM91 (142.9 mAh g^−1^). In the second rate test, as the current density increased, R-NM91@LTO again exhibited higher capacity than C-NM91. These results highlight that the relithiation and recrystallization processes endow R-NM91@LTO with superior structural integrity, which directly contributes to its outstanding high-rate performance.

To further demonstrate the advantages of regenerated R-NM91@LTO, its long-term cycling stability was first evaluated at 25 °C under 1C in the voltage range of 2.8–4.3 V (Figure 4e). Clearly, R-NM91@LTO still maintains a discharge capacity of 161.5 mAh g^−1^ after 300 cycles, corresponding to 84.3% of its initial capacity. R-NM91 shows an initial capacity of about 190.3 mAh g^−1^ and retains about 146.2 mAh g^−1^ after 300 cycles, corresponding to a retention of about 76.8%. Under the same conditions, C-NM91 retains only 136.8 mAh g^−1^, with a corresponding capacity retention of 70.6%, whereas S-NM91 starts from only 132 mAh g^−1^ and undergoes even more severe decay. In addition to its lower capacity retention, C-NM91 also shows progressively aggravated voltage decay during cycling, indicating accelerated polarization and significant resistance accumulation (Figure 4f). The charge–discharge curves of R-NM91 overlap better than those of C-NM91, showing slower polarization growth and improved capacity retention (Figure 4g). By comparison, the charge–discharge curves of R-NM91@LTO are almost completely overlapped, with negligible voltage plateau decay, minimal polarization, and nearly no capacity loss, directly confirming its excellent structural stability (Figure 4h). Because the layered structure of the severely degraded spent sample is seriously damaged, its obvious electrochemical plateau can hardly be identified (Figure S7). Overall, compared with the traditional polycrystalline structure, the single-crystal structure achieves comprehensive improvements in capacity, cycling stability, and voltage retention, effectively addressing grain boundary cracking and severe side reactions in polycrystalline materials. The recycling process successfully reconstructs spent polycrystalline cathodes into high-performance single-crystal cathodes, while the synergistic effect of the LTO coating and radial single-crystal structure establishes a stable cathode–electrolyte interphase (CEI), suppresses transition-metal dissolution and electrolyte decomposition, and ultimately enables R-NM91@LTO to achieve exceptionally high capacity retention and excellent voltage stability. This provides a feasible approach for developing high-energy-density and long-life ternary cathode materials.

**Figure 5:**
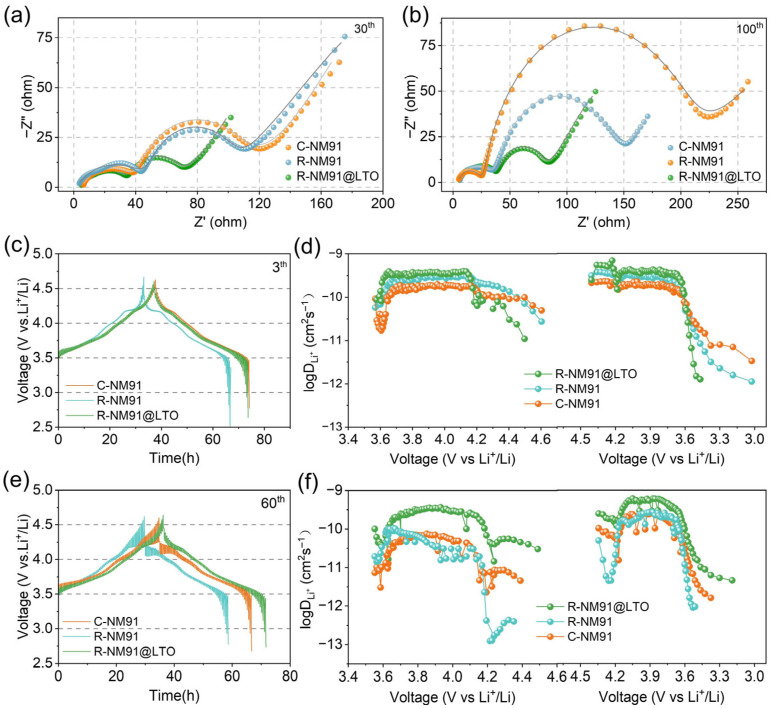
EIS impedance spectra of C-NM91, R-NM91 and R-NM91@LTO after (**a**) 10 cycles and (**b**) 100 cycles, GITT plots obtained during the (**c**) 3rd and (**e**) 60th charge/discharge cycles, and the (**d**,**f**) corresponding *D*_Li_^+^ values.

Electrochemical impedance spectroscopy (EIS) and galvanostatic intermittent titration technique (GITT) analyses were further conducted to validate the excellent electrochemical reversibility and lithiation/delithiation kinetics of regenerated R-NM91@LTO. EIS plots and corresponding fitted profiles taken from the 10th (Figure 5a) and 100th (Figure 5b) along with the fitted results (Table S2) derived from the equivalent circuit model (Figure S8), reveal after 10 cycles, all three samples exhibit relatively low interfacial resistance, among which R-NM91@LTO shows the lowest charge transfer resistance (*R*_ct_) of only 29.42 Ω, lower than that of R-NM91 (46.45 Ω) and C-NM91 (53.76 Ω). After 100 cycles, the resistance of all samples increases to varying degrees, but the difference among the three becomes much more pronounced. C-NM91 exhibits the most severe increase in resistance, from 53.76 Ω to 164.5 Ω. The resistance growth of R-NM91 is much smaller than that of C-NM91, increasing from 46.45 Ω to 88.8 Ω. In contrast, R-NM91@LTO shows the smallest increase after cycling, from 29.42 Ω to 37.39 Ω. These results clearly demonstrate that the synergistic effect of the LTO coating and single-crystal structure successfully establishes a stable cathode–electrolyte interphase, prevents electrolyte corrosion of the active material, suppresses transition-metal dissolution and interfacial side reactions, and thus maintains an extremely low charge transfer resistance during prolonged cycling [49,50].

To further support this conclusion, GITT curves were collected during the 3rd (Figure 5c) and 60th (Figure 5e) charge–discharge cycles at 1C between 2.8 and 4.6 V. The lithium-ion diffusion coefficients (*D*_Li_^+^) calculated from the GITT curves provide deeper insight into the kinetics of the electrochemical process. The key distinction is that R-NM91@LTO combines the dual advantages of a single-crystal structure and LTO coating, whereas C-NM91 has a conventional polycrystalline structure. This structural difference directly affects their electrochemical behavior and long-term stability [51]. During the 3rd cycle, R-NM91@LTO exhibits the highest *D*_Li_^+^ value among the tested materials, outperforming both R-NM91 and C-NM91 (Figure 5d). The continuous Li^+^ transport pathway provided by the single-crystal structure, together with the reduced interfacial charge transfer resistance induced by the LTO coating, synergistically optimizes the initial ion transport kinetics [52]. By contrast, polycrystalline C-NM91, with its abundant grain boundaries and significant scattering effect, exhibits the lowest *D*_Li_^+^. In subsequent cycles, the difference becomes increasingly pronounced. By the 50th cycle, C-NM91 shows markedly accelerated voltage decay accompanied by a sharp drop in *D*_Li_^+^. Although R-NM91 is improved relative to C-NM91 by virtue of its single-crystal structure, the absence of LTO protection allows interfacial side reactions to gradually consume active sites, causing *D*_Li_^+^ to drop sharply in the high-voltage region and severely deteriorating ion transport efficiency (Figure 5f). In contrast, owing to the synergistic combination of the single-crystal framework and LTO coating, R-NM91@LTO effectively resists lattice phase transitions and particle microcrack formation during long-term cycling, while the LTO layer further isolates the cathode from direct contact with the electrolyte and suppresses interfacial side reactions and structural strain. As a result, even after cycling, it still maintains a clear and flat charge–discharge plateau and a relatively high *D*_Li_^+^ level, fully preserving the structural integrity and kinetic stability of the regenerated material. These findings clearly demonstrate the advantages of the single-crystal structure and LTO coating.

**Figure 6:**
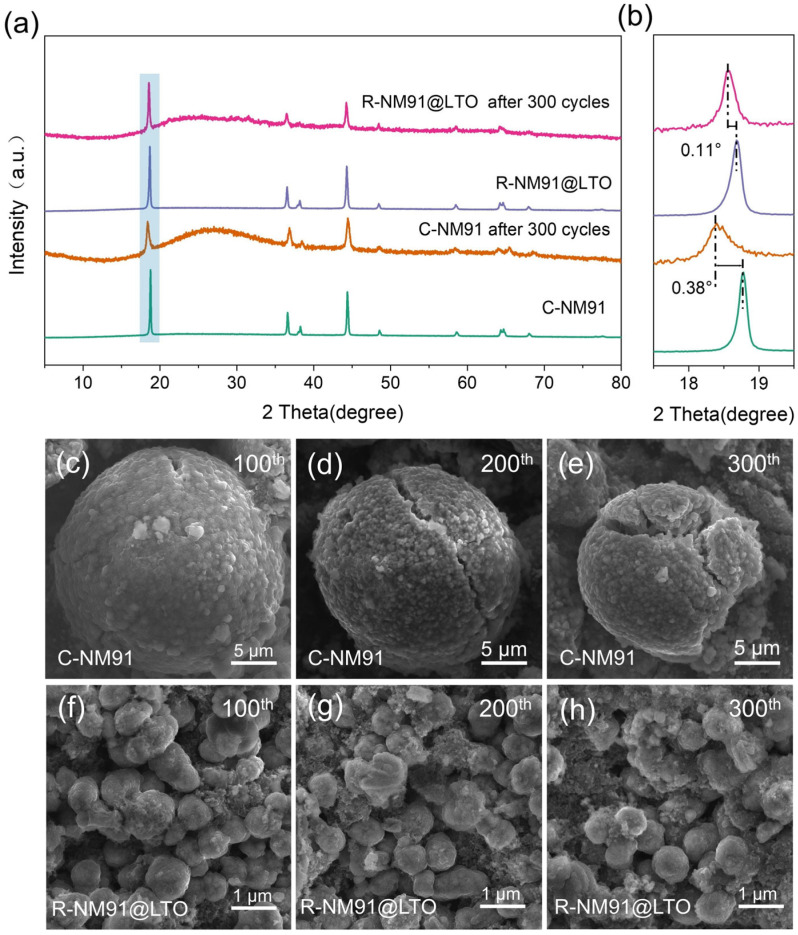
(**a**) XRD patterns of the electrode sheets before and after cycling; (**b**) a magnified view of the (003) diffraction peak. FESEM images of the electrode pellets during the 100th, 200th and 300th cycles for (**c**–**e**) C-NM91 and (**f**–**h**) R-NM91@LTO.

Figure 6a shows the XRD patterns of C-NM91, R-NM91@LTO, and their corresponding samples after 300 cycles. All diffraction peaks can be well indexed to the *α*-NaFeO_2_-type layered structure (*R-3m* space group), with no obvious impurity phases detected, indicating that both materials essentially maintain the layered crystal structure after long-term cycling. However, comparison before and after cycling reveals that the characteristic peaks of commercial polycrystalline C-NM91 become significantly weaker and much broader after 300 cycles, reflecting severe structural degradation, lattice distortion, and loss of layered ordering during prolonged cycling. In contrast, the diffraction peaks of regenerated R-NM91@LTO remain sharp and intense after 300 cycles, with a peak profile highly consistent with that of the fresh sample, directly demonstrating its excellent structural stability. An enlarged view of the (003) peak further quantifies the structural evolution. After cycling, the (003) peak of C-NM91 shifts by 0.38° and broadens significantly, indicating severe lattice expansion and structural stress. By comparison, the (003) peak of R-NM91@LTO shifts by only 0.11° with almost no broadening, indicating that its lattice remains highly stable during long-term cycling (Figure 6b) [53]. This confirms that the synergistic effect of the LTO coating and single-crystal structure effectively suppresses phase transitions, stress accumulation, and structural collapse during cycling. Overall, the XRD results show that R-NM91@LTO fundamentally addresses the structural degradation problem of conventional polycrystalline NCM through single-crystal reconstruction and interfacial coating modification, thereby providing solid structural support for its excellent long-term electrochemical performance.

To directly visualize structural degradation, the cycled cathodes recovered from half-cells after different cycle numbers within 2.8–4.3 V were further examined in detail. FESEM images illustrating the morphological evolution of commercial polycrystalline C-NM91 and regenerated single-crystal R-NM91@LTO after 100, 200, and 300 cycles clearly reveal the difference in structural stability between the two materials during long-term cycling. For C-NM91, obvious microcracks and structural damage already appear on the particle surface after 100 cycles (Figure 6c), indicating the initial breakdown of particle integrity. After 200 cycles, the cracks further expand and penetrate the particles, accompanied by pronounced stress-induced fracture and aggravated grain boundary separation (Figure 6d). After 300 cycles, the particles undergo severe fragmentation and pulverization, and the structure collapses completely (Figure 6e). In contrast, R-NM91@LTO still retains an intact single-crystal particle morphology after 100 cycles, with a dense and smooth surface and no obvious cracks or structural damage (Figure 6f). After 200 cycles, the particle morphology and size remain essentially unchanged, with only slight traces of interfacial side reactions on the surface (Figure 6g). Even after 300 cycles, the particles still maintain a complete single-crystal structure without obvious fracture or pulverization (Figure 6h), fully demonstrating that the synergistic effect of the single-crystal structure and LTO coating effectively suppresses volume expansion, stress accumulation, and electrolyte corrosion during cycling, thereby ensuring long-term stability at the microstructural level. Overall, the FESEM observations confirm that R-NM91@LTO fundamentally resolves the cycling-induced structural degradation of conventional polycrystalline NCM through structural reconstruction and interfacial modification, thereby providing robust microstructural support for its excellent electrochemical performance.

## 4. Conclusions

In summary, this study proposes an innovative direct regeneration strategy integrating acid etching, hydrothermal relithiation, and short-time annealing, together with the simultaneous construction of a Li_4_Ti_5_O_12_ (LTO) surface coating layer, thereby effectively addressing the long-standing challenges of difficult relithiation and poor structural repair in spent high-nickel layered cathodes. This method is mild, simple, and robust, and enables the directional transformation of severely degraded Ni-rich polycrystalline cathodes into structurally stable and highly crystalline single-crystal R-NM91@LTO, achieving both structural upgrading and substantial enhancement in electrochemical performance. Structural characterization shows that Li/Ni cation mixing is significantly reduced, layered ordering and lattice integrity are markedly restored, and a uniform and dense LTO coating layer is formed on the surface, effectively suppressing interfacial side reactions and the propagation of particle microcracks. Electrochemical tests confirm that, compared with commercial polycrystalline cathodes, the regenerated single-crystal cathode exhibits lower polarization, faster lithium-ion diffusion kinetics, and significantly enhanced rate capability and long-term cycling stability, delivering a capacity retention of 84.3% after 300 cycles at 1C, while structural phase transitions and lattice distortion are effectively suppressed. This strategy also shows good regeneration performance for various high-nickel ternary systems and offers outstanding advantages over pyrometallurgical and hydrometallurgical recycling in terms of low energy consumption, low emissions, and high added value. Therefore, this work provides an efficient and feasible technical route for the green regeneration and high-value utilization of spent high-nickel ternary cathodes and is of great significance for advancing sustainable recycling of spent lithium batteries and the development of high-performance energy storage materials.

## Data Availability

The original contributions presented in this study are included in the article/Supplementary Materials. Further inquiries can be directed to the corresponding author.

## Supplementary Materials

The following supporting information can be downloaded at: https://www.mdpi.com/article/10.3390/nano16100585/s1, Figure S1: Low and high magnification FESEM images of (a,b) E-NM91; Figure S2: Low and high magnification FESEM images of (a,b) C-NM91; Figure S3: Ni 2p spectra of R-NM91; Figure S4: O 2p spectra of R-NM91; Figure S5: Images of S-NM91 at different CV scan rates; Figure S6: Images of R-NM91 at different CV scan rates; Figure S7: Voltage profiles of S-NM91 within 2.8–4.3 V at 25 °C; Figure S8: Equivalent circuit used to fit the data in the Nyquist plot; Table S1: Lattice parameters calculated from Rietveld refinement of XRD data of the S-NM91, C-NM91 and R-NM91@LTO; Table S2: EIS fitting parameters for C-NM91, R-NM91 and R-NM91@LTO samples at 10th and 100th cycles; Table S3: Based on the ICP-OES data, the stoichiometric element ratios for different samples have been summarised.
