# Supplementary material for: Efficient Regeneration of Degraded LiNi0.9Mn0.1O2 by Acid Etching–Hydrothermal Relithiation Coupled with Li4Ti5O12 Coating
Source: Nanomaterials (Basel). 2026 May 11;16(10):585. doi: 10.3390/nano16100585 (PMC13209473; doi:10.3390/nano16100585)
Supplement: Supplementary file 1 [file nanomaterials-16-00585-s001.zip › nanomaterials-4293843-supplementary.pdf]

nanomaterials

Supporting Information

Upcycling Spent Polycrystalline $LiNi_{0.9}Mn_{0.1}O_2$ into Single-Crystal Cathodes by Acid Etching–Hydrothermal Relithiation Coupled with $Li_4Ti_5O_{12}$ Coating

Jiwei Hao, Longwei Liang *, Jiawei Mu, Zhenyuan Xie, Hongqiang Xi, Linrui Hou and Changzhou Yuan *

School of Materials Science & Engineering, University of Jinan, Jinan 250022, China; haojw@stu.ujn.edu.cn (J.H.); mujw@stu.ujn.edu.cn (J.M.); xiezhenyuan@stu.ujn.edu.cn (Z.X.); xihq@stu.ujn.edu.cn (H.X.); mse_houlr@ujn.edu.cn (L.H.)

* Correspondence: mse_lianglw@ujn.edu.cn (L.L.); mse_yuancz@ujn.edu.cn or ayuancz@163.com (C.Y.)

Supplementary figures

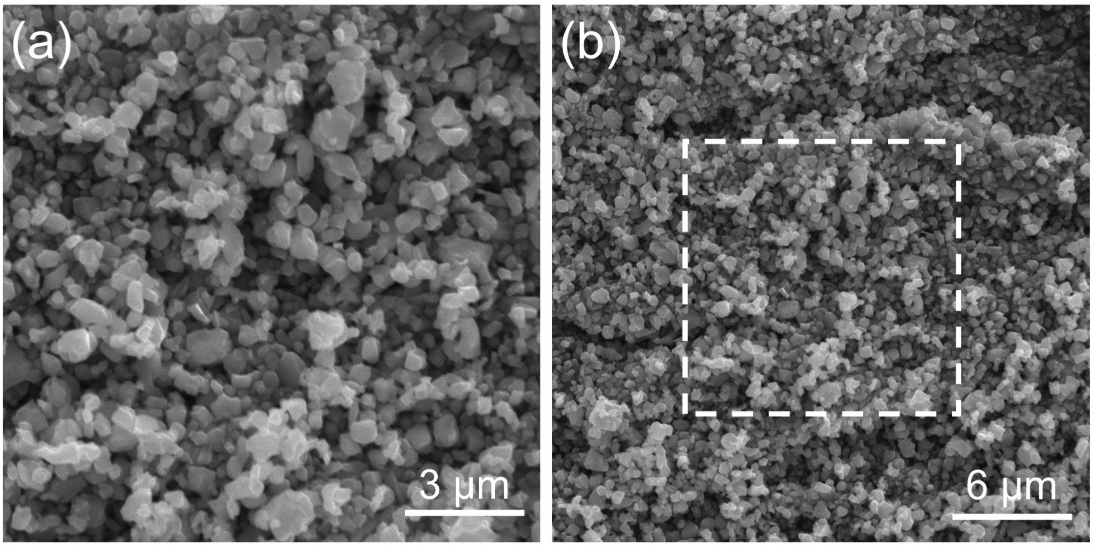

Figure S1. Low and high magnification FESEM images of (a, b) E-NM91.

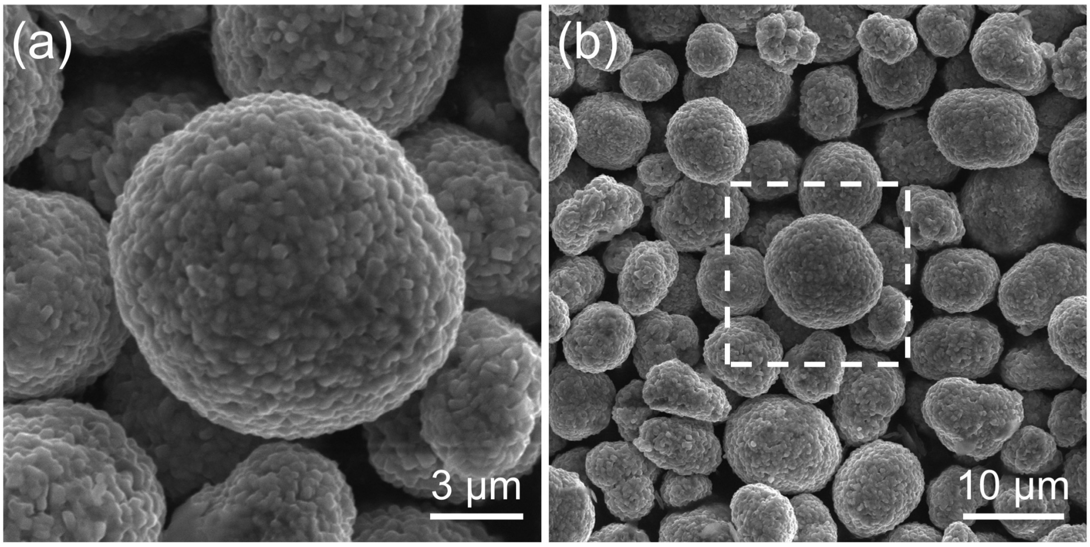

Figure S2. Low and high magnification FESEM images of (a, b) C-NM91.

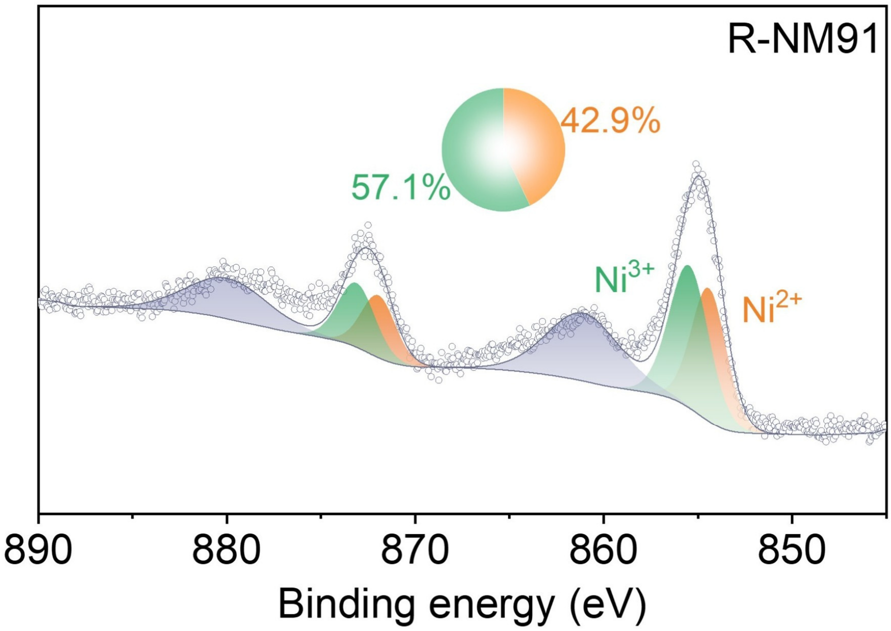

Figure S3. Ni 2p spectra of R-NM91.

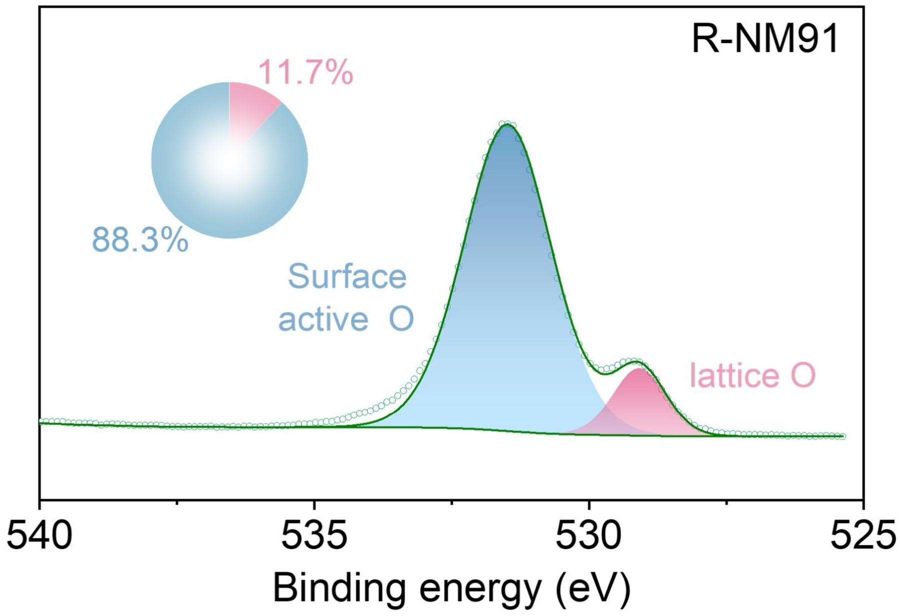

Figure S4. O 2p spectra of R-NM91.

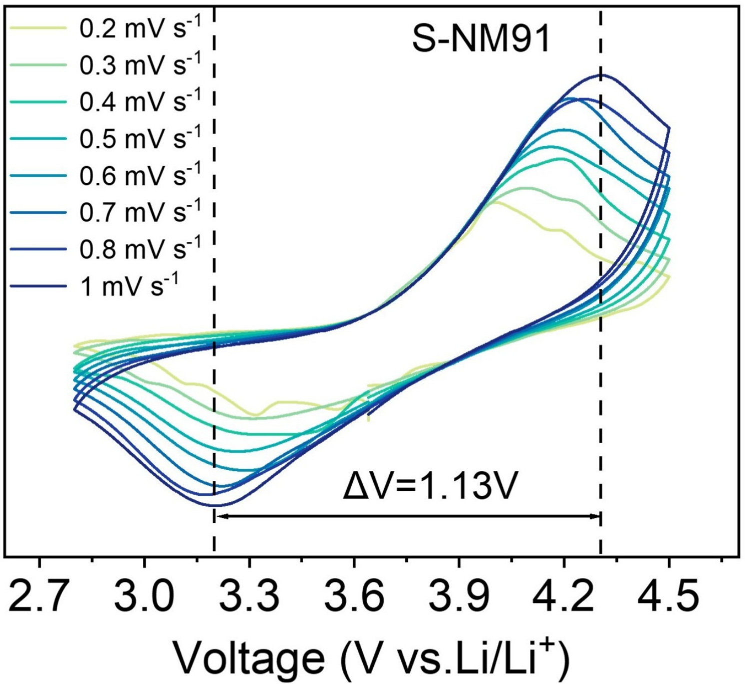

Figure S5. Images of S-NM91 at different CV scan rates.

https://doi.org/10.3390/nano16100585

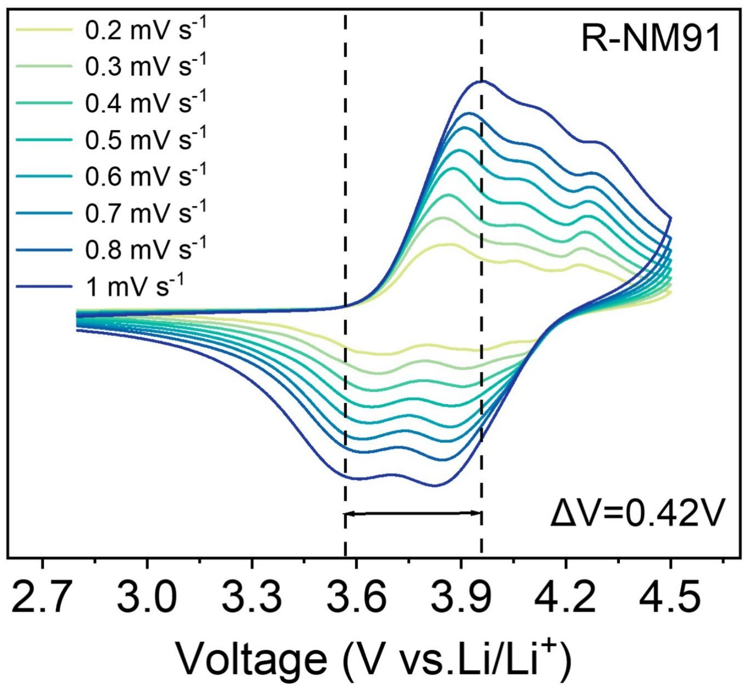

Figure S6. Images of R-NM91 at different CV scan rates

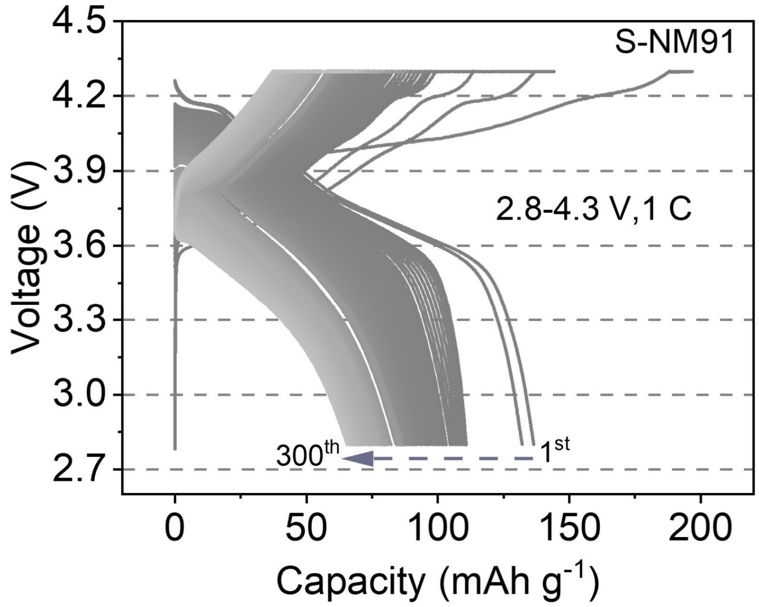

Figure S7. Voltage profiles of S-NM91 within 2.8 − 4.3 V at 25 °C .

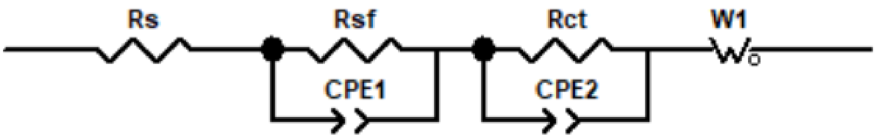

Figure S8. Equivalent circuit used to fit the data in the Nyquist plot.

https://doi.org/10.3390/nano16100585

Supplementary tables

Table S1. Lattice parameters calculated from Rietveld refinement of XRD data of the S-NM91, C-NM91 and R-NM91@LTO.

Samples	*a* (Å)	*c* (Å)	V (Å^3)	Ni^{2+} in Li^{+} site/%	R_{wp} (%)	R_p (%)
S-NM91	2.8662992	14.412479	102.545	9.97%	5.37%	3.99%
C-NM91	2.878590	14.195352	101.868	3.49%	6.36%	4.73%
R-NM91@LTO	2.879595	14.216388	101.989	4.11%	6.45%	4.57%

Table S2. EIS fitting parameters for C-NM91, R-NM91 and R-NM91@LTO samples at 10th and 100th cycles.

sample / cycle	C-NM91 (R_f/R_{ct}, Ω)	R-NM91 (R_f/R_{ct}, Ω)	R-NM91@LTO (R_f/R_{ct}, Ω)
10th	30.15/53.76	48.49/46.45	22.7/29.42
100th	20.5/164.5	29.15/88.88	36.13/37.39

Table S3. Based on the ICP-OES data, the stoichiometric element ratios for different samples have been summarised.

Samples	Li (Molar Ratio)	Ni (Molar Ratio)	Mn (Molar Ratio)
S-NM91	0.881	0.892	0.108
C-NM91	1.011	0.895	0.105
R-NM91@LTO	1.007	0.891	0.109

https://doi.org/10.3390/nano16100585
